# Prognostic Value of B7H4 Expression in Patients with Solid Cancers: A Systematic Review and Meta-Analysis

**DOI:** 10.3390/ijms25095045

**Published:** 2024-05-06

**Authors:** Miriam Dawidowicz, Agnieszka Kula, Sylwia Mielcarska, Elżbieta Świętochowska, Dariusz Waniczek

**Affiliations:** 1 Department of Oncological Surgery, Faculty of Medical Sciences in Zabrze, Medical University of Silesia, 41-808 Katowice, Poland; d201070@365.sum.edu.pl; 2 Department of Medical and Molecular Biology, Faculty of Medical Sciences in Zabrze, Medical University of Silesia, 41-800 Zabrze, Poland; d201109@365.sum.edu.pl (S.M.); eswietochowska@365.sum.edu.pl (E.Ś.)

**Keywords:** B7H4, cancer prognosis, immune checkpoint, solid cancers

## Abstract

V-set domain-containing T-cell activation inhibitor 1 (aliases VTCN1, B7H4) participates in tumour immune escape by delivering inhibitory signals to T cells. The purpose of this article was to assess the B7H4 prognostic value in solid cancers. Three databases were searched for relevant articles. The main endpoints were overall survival (OS), disease-specific survival (DSS), progression-free survival (PFS), recurrence-free survival (RFS), and disease-free survival (DFS). Appropriate hazard ratios (HRs) were pooled. The R studio software (version 4.0.3) was used for data analysis. Thirty-one studies met the inclusion criteria. High expression of B7H4 was associated with worse OS (HR = 1.52, 95% CI: 1.37–1.68) but not with DSS (HR = 1.14, 95% CI: 0.49–2.63), RFS (HR = 1.77, 95% CI: 0.75–4.18), DFS (HR = 1.29, 95% CI: 0.8–2.09), or PFS (HR = 1.71, 95% CI: 0.91–3.2) in patients with solid cancers. High expression of B7H4 is associated with a poorer prognosis in patients with solid cancers. B7H4 is a promising prognostic biomarker and immunotherapeutic target for various solid cancers because of its activity in cancer immunity and tumourigenesis.

## 1. Introduction

### 1.1. B7H4 Expression and Function

B7H4 is a member of the B7 immune checkpoint family. The immune checkpoints are regulators of the immune system and are crucial for self-tolerance [1]. B7H4 is a coinhibitory ligand that exerts its function by suppressing the T cell effector function, while its molecule interaction on T cells remains unknown. B7H4 is broadly overexpressed in human cancers, including lung, liver, kidney, ovary, stomach, skin, pancreas, colorectal, and breast cancers, and is exploited by tumour in order to evade immune surveillance [2,3,4,5,6].

### 1.2. B7H4 in Solid Cancers

B7H4 is predominantly expressed on antigen-presenting cells (APCs) and tumour cells. Its heightened activity in cancer is associated with the increased infiltration of immunosuppressive cells and elevated production of regulatory T cells, leading to a reduced proliferation and effector function of CD4^+^ and CD8^+^ T cells [7]. Contrary to PD-L1, which is expressed in about 30% or even fewer patients and is associated with immunologically “hot” tumours, the expression of B7H4 marks a “cold” environment. The researchers observed, in some cases, a resistance to immune checkpoint inhibitor therapy, which mainly targets PD-1/PD-L1 and CTLA4 pathways [8,9,10]. This effect might be possibly explained by the activation of alternative immune checkpoints, e.g., B7H4. Aberrant B7x expression is associated with tumour necrosis, stage, grade, epithelial–mesenchymal transition (EMT), and survival outcomes such as progression free survival (PFS) and overall survival (OS), making it a potential blood biomarker in many cancers [11,12,13,14]. B7H4 has a soluble form called sB7H4 that can be detected in blood serum of cancer patients [15,16,17]. B7H4 expression is significantly limited in healthy tissues [18]. Elevated levels of sB7H4 were detected in the sera of gastric, hepatocellular, and renal cancer patients. In those studies, patients with higher levels of sB7H4 had a significantly shorter OS and higher probability of recurrence [15,16,17]. However, there is a very limited number of studies that evaluate the association of sB7H4 with cancer prognosis to conduct the meta-analysis. Instead, we performed the most comprehensive and up-to-date meta-analysis of B7H4 expression assessed by the immunohistochemistry method (IHC) and prognostic outcomes among solid cancers. Furthermore, we assessed all available surrogate endpoints, such as DFS, PFS, and RFS. Nowadays, B7H4 is extensively studied as a therapeutic target due to its role in immune system suppression in tumourigenesis [19,20,21]. Its expression is also distinct from the PD-1/PD-L1 pathways; thus, it provides the chance for effective therapies for the vast majority of cancer patients with B7H4-positive tumours [22,23]. Besides its therapeutic role, B7H4 might be a potentially good screening protein as it is secreted into the blood stream by cancer cells and paracancerous tissue, and it is mostly absent in healthy cells. On the other hand, it is not useful as a cancer-specific biomarker due to its overexpression in a variety of cancers. Study results also suggest that it might play a prognostic, predictive, and potentially monitoring role in cancers. This knowledge may be applied in the future in clinical decision making.

### 1.3. The Aim of the Study

As B7H4 is a relatively new immune checkpoint, its prognostic value in cancer prognosis has yet to be estimated. Currently available meta-analyses that tackle the topic of B7H4’s prognostic role were mostly conducted over 5 years ago. Moreover, as the number of studies was limited at that time, they are based on results from mixed methods of estimating B7H4 expression (ELISA, IHC) and sources of B7H4 expression (tissue, homogenates, blood sera) [24]. Additionally, some included different methods of prognosis estimation, such as the odds ratio (OR), rate ratio (RR), and hazard ratio (HR), to conduct the meta-analysis [25]. Furthermore, most studies were focused on the overall survival (OS) parameter, and two studies also evaluated disease-free survival (DFS) [25,26]. The disease-specific survival (DSS), progression-free survival (PFS), and recurrence-free survival (RFS) parameters have not yet been evaluated in meta-analyses. Therefore, we conducted a meta-analysis with one method of estimation of B7H4 expression (IHC) and prognosis (HR) to evaluate the prognostic value of B7H4 in parameters such as OS, DSS, DFS, PFS, and RFS among solid cancers.

## 2. Results

### 2.1. Search Results and Study Characteristics

**Figure 1:**
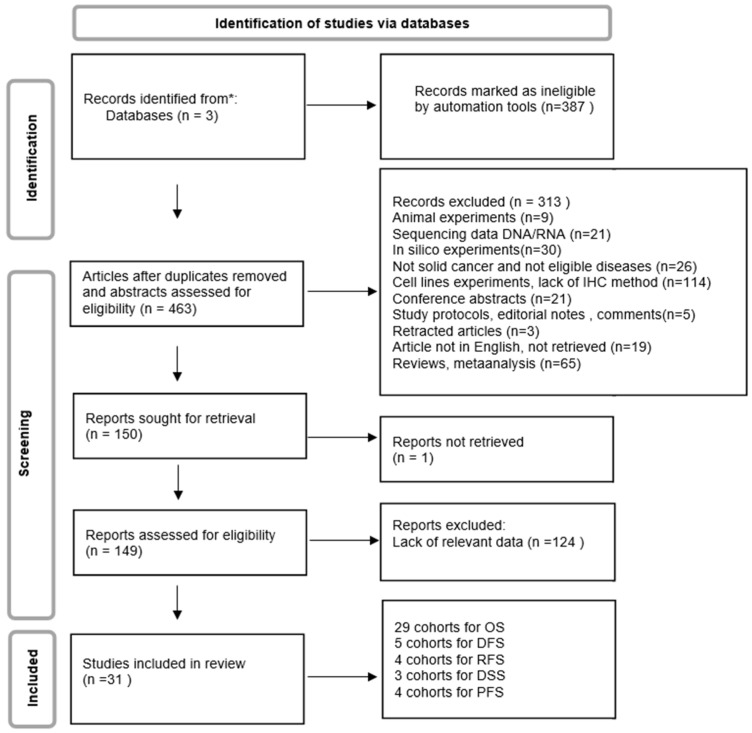
Study selection flowchart.

A flow diagram showing our literature search and screening strategy is presented in Figure 1. A total of 850 articles were initially identified through database research. After removing 387 studies by applying automatic tools, the remaining 463 records were screened by reading the titles and abstracts. Further, 313 studies were excluded. One hundred and forty-nine studies were evaluated for eligibility, and, finally, 31 articles were included in the meta-analysis. The basic characteristics of the included studies are shown in Table 1. The studies were published between 2007 and 2023. They consisted of the following cancer types: OS—osteosarcoma [27], Pca—prostate cancer [28,29], CvC—cervical cancer [30,31], CCA—cholangiocarcinoma [13,32], PDAC—pancreatic ductal adenocarcinoma [33,34,35,36], UCC—urothelial cell carcinoma [36], HNSCC—head and neck squamous cell carcinoma [37,38], OC—ovarian cancer [39], ESCC—esophageal squamous cell carcinoma [28,40], BC—breast cancer [41,42], GC—gastric cancer [43,44], ECC—endometrial cancer [45], NSCLC—non-squamous cell lung carcinoma [46,47], RCC—renal cell carcinoma [48], and CRC—colorectal cancer [39,49,50,51,52]. The sample sizes ranged from 37 to 996, with a total of 6357 patients. B7H4 expression was measured by IHC in all cohorts. HRs and the corresponding 95% CIs of the assessed parameters were obtained by the multivariate analysis in 20 cohorts and univariate analysis or Kaplan–Meier curves in 11 cohorts. The NOS scores of all these studies were between 6 and 8 points, except for one article, which scored 5 points (Supplementary Material Table S4).

### 2.2. Results of Overall Survival Meta-Analysis

**Figure 2:**
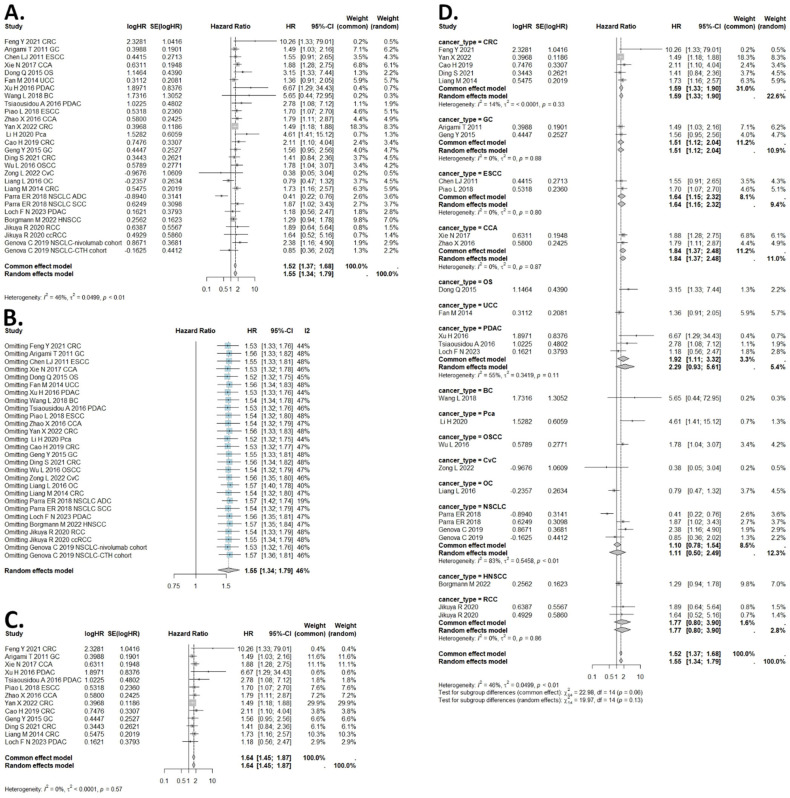
Forest plots of studies evaluating HRs of high B7H4 expression and overall survival in solid tumours and the subgroup analysis. (**A**) Forest plot of overall survival among all solid tumours. (**B**) One−leave meta−analysis for investigating the effects of particular studies on the association between B7H4 expression and overall survival in solid tumours. (**C**) Forest plot of overall survival in gastrointestinal cancers. (**D**) Forest plot of overall survival in cancer type subgroups.

In total, 29 cohorts were qualified to assess the relation between B7H4 and OS, including both univariate and multivariate analyses. In the studies that reported both univariate and multivariate HRs, we used multivariate to diminish the risk of bias. The I (2) value was less than 50%, and the *p* value was less than 0.01, so the common effect model was used in the OS comparison. The results of the overall survival in solid tumours showed that high expression of B7H4 was associated with shorter OS (common effect model HR = 1.52, 95% CI: 1.37–1.68) (Figure 2A). We performed subgroup analysis to explore the potential factors that may cause heterogeneity. We classified the included cohorts and conducted subgroup analysis based on the cancer type, sample size, final score assessed in IHC analysis, and analysis method (Figure 2C,D, Supplementary Materials Figure S3). Subgroup analysis of gastrointestinal tumours revealed that B7H4 overexpression was correlated with poor OS, with an HR of 1.59, 95% CI: 1.27–1.98 (Figure 2C). The subgroup analysis by cancer type further confirmed the association of high B7H4 expression with shorter OS in patients with CCA (HR = 1.84, 95% CI: 1.37–2.48), ESCC (HR = 1.64, 95% CI: 1.15–2.32), CRC (HR = 1.59, 95% CI: 1.33–1.90), and GC (HR = 1.51, 95% CI: 1.12–2.04) but not in patients with PDAC (HR = 2.29, 95% CI: 0.93–5.61), RCC (HR = 1.77, 95% CI: 0.8–3.9), and NSCLC (HR = 1.11, 95% CI: 0.50–2.49) (Figure 2D). In addition, the subgroup analysis according to cancer type showed that there was significant heterogeneity within the PDAC and NSCLC subgroups (Figure 2D). When the subgroup analysis was performed according to the analysis method and final IHC score, the results changed. OS estimated by the univariate method did not support the B7H4 impact on survival unlike the multivariate method (HR = 1.26, 95% CI: 0.96–1.67 vs. HR = 1.61, 95% CI: 1.48–1.91) (Supplementary Materials Figure S3A). Similarly, the method of estimation of B7H4 expression impacted the results. The studies that assessed B7H4 IHC score with a cut-off > 3 had low heterogeneity and supported its association with worse OS (HR = 1.64, 95% CI: 1.41–1.90) (Supplementary Materials Figure S3C). Sample size did not change HRs significantly (Supplementary Materials Figure S3B).

### 2.3. Results of DSS, PFS, RFS, and DFS Meta-Analysis

**Figure 3:**
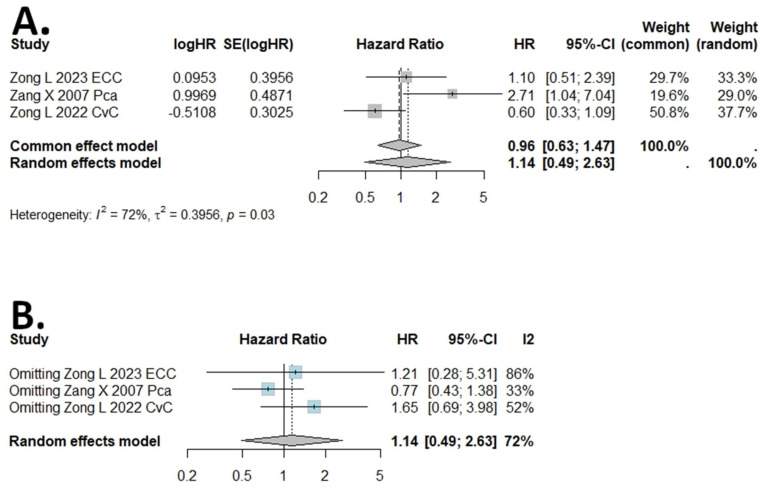
(**A**) Forest plot of studies evaluating HRs of high B7H4 expression and disease−specific survival (DSS) in solid tumours. (**B**) One−leave meta−analysis for investigating the effects of particular studies on the association between B7H4 expression and DSS in solid tumours.

**Figure 4:**
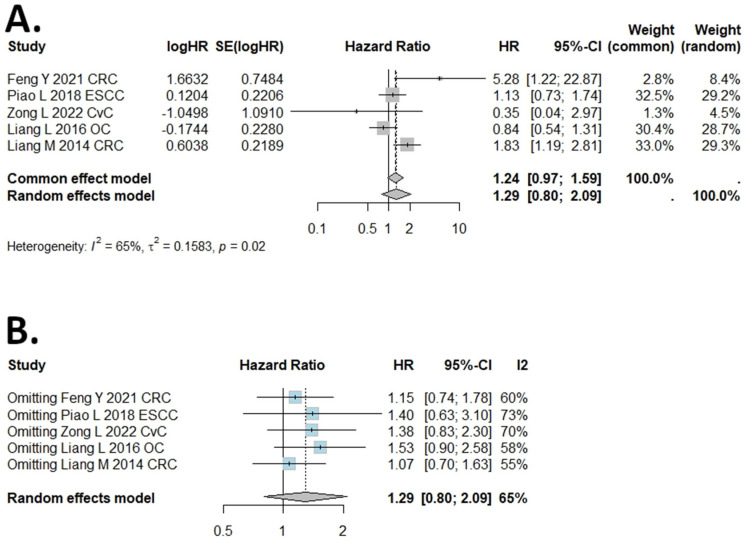
(**A**) Forest plot of studies evaluating HRs of high B7H4 expression and disease−free survival (DFS) in solid tumours. (**B**) One-−leave meta−analysis for investigating the effects of particular studies on the association between B7H4 expression and DFS in solid tumours.

**Figure 5:**
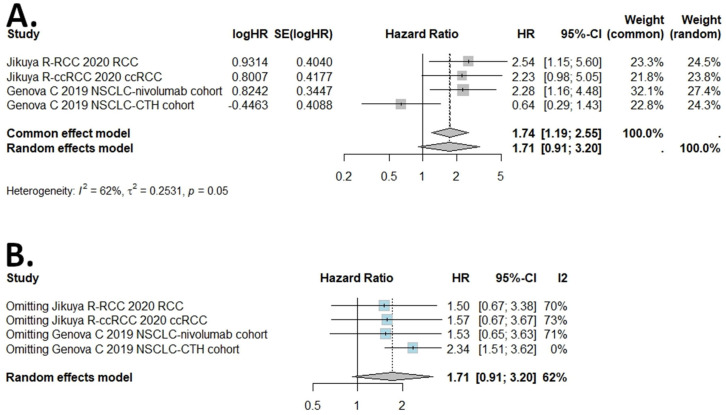
(**A**) Forest plot of studies evaluating HRs of high B7H4 expression and progression−free survival (PFS) in solid tumours. (**B**) One−leave meta−analysis for investigating the effects of particular studies on the association between B7H4 expression and PFS in solid tumours.

**Figure 6:**
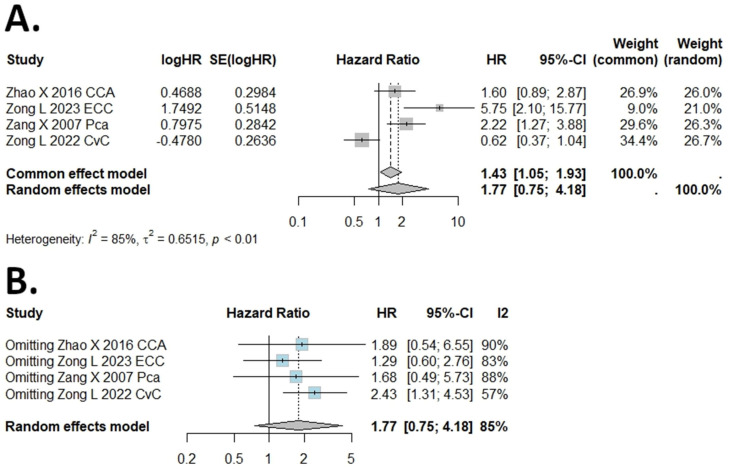
(**A**) Forest plot of studies evaluating HRs of high B7H4 expression and recurrence−free survival (RFS) in solid tumours. (**B**) One−leave meta−analysis for investigating the effects of particular studies on the association between B7H4 expression and RFS in solid tumours.

The I (2) value was more than 50%, and the *p* value was less than 0.05, so the random-effects model was used in the comparison of DSS, DFS, PFS, and RFS (Figure 3, Figure 4, Figure 5 and Figure 6). The pooled results of the meta-analysis showed that a high expression of B7H4 was not associated with shorter DSS (HR = 1.14, 95% CI: 0.49–2.63), RFS (HR = 1.77, 95% CI: 0.75–4.18) (Figure 3A and Figure 6A, respectively), DFS (HR = 1.29, 95% CI: 0.8–2.09), or PFS (HR = 1.71, 95% CI: 0.91–3.2) (Figure 4A and Figure 5A, respectively) in the patients with solid cancers than a low expression of B7H4. In the sensitivity analysis, the PFS and RFS results would change if the Genova C 2019 NSCLC CTH cohort [47] and Zong L. 2022 [31] were omitted, respectively (Figure 5B and Figure 6B).

The *p* values of Begg’s test and Egger’s test for OS were above 0.05, which indicated no significant publication bias. Zong L. 2023, Piao L. 2018, Jikuya R. 2019 ccRCC cohort, and Zhao X. 2016 contributed the most to the overall heterogeneity in DSS, DFS, PFS, and RFS, respectively.

## 3. Methods

### 3.1. Data Sources and Search Strategy for Meta-Analysis

This systematic review and meta-analysis were performed according to the Preferred Reporting Items for Systematic Reviews and Meta-Analysis (PRISMA) guidelines [54]. Embase (https://www.embase.com/ accessed on 1 July 2023), PubMed (https://pubmed.ncbi.nlm.nih.gov/ accessed on 10 July 2023), and the Cochrane Library (https://www.cochrane-library.com/ accessed on 1 August 2023) were searched for articles. The retrieval time was from the inception to 17 September 2023. This review was registered on the PROSPERO platform (CRD42023414613). The search strategy is described in detail in Supplementary Materials Tables S1–S3.

### 3.2. Inclusion and Exclusion Criteria

Inclusion criteria included patients diagnosed with solid cancer before enrolment, randomised controlled trials (RCTs) or observational studies, sufficient data about B7H4 expression evaluated by the immunohistochemical method (IHC), a clinical outcome with a provided hazard ratio (HR), or, in cases where the HR was not provided, the Kaplan–Meier curve with a number at risk table was accepted. The outcomes included overall survival (OS), disease-specific survival (DSS), disease-free survival (DFS), recurrence-free survival (RFS), and progression-free survival (PFS) (OS, DSS, DFS, PFS, RFS definitions: Supplementary Material Table S5). The exclusion criteria included a lack of sufficient data, non-solid and nervous system cancer, case reports, sequencing data studies, animal experiments, studies based on TCGA and other online available repository to avoid the duplication of data, meta-analyses, network meta-analyses, reviews, conference presentations, or study protocols.

### 3.3. Study Selection and Data Extraction

Two review authors (Miriam Dawidowicz and Agnieszka Kula) independently reviewed the titles and study abstracts with potential eligibility. The full texts of eligible studies were downloaded for further assessment. Three authors (Miriam Dawidowicz, Agnieszka Kula, Sylwia Mielcarska) independently extracted the following data: basic information, such as the first author, publication year, sample size, country, and study design; characteristics of patients, type, and stage of cancer; more detailed information regarding the clinical outcomes; information of cancer treatment, details about B7H4 expression location and cut-off value determining high expression, HR estimation method (univariate or multivariate analysis), and HR. Any disagreement was resolved by group discussion and consensus. We excluded results reported in only one study. If the study did not report an HR, but a survival curve with a number at risk table was published, the HR values were reconstructed using WebPlotDigitizer v4.7 and an algorithm was developed by Guyot P in R Studio [55]

### 3.4. Statistical Analysis for Meta-Analysis

To conduct all analyses, we used R software (version 4.0.3). For estimating the HR, multivariate analysis models were used; if not provided in the articles, univariate models were used. To estimate the heterogeneity, the chi-square Cochran’s Q-test and Higgins I2 statistics were performed. I2 values were interpreted as follows: 25–50%—low heterogeneity, 50–75%—moderate heterogeneity, above 75%—high heterogeneity according to J. P. Higgins and Thompson [56]. A fixed-effects model was used to pool the value of the HR and 95% confidence interval if I2 < 50% and *p* value > 0.05, indicating the lack of substantial heterogeneity. The random-effects model was applied when the significant heterogeneity was determined. In order to test the effect of the exclusion of one study each time, sensitivity analyses were performed. The publication bias assessments were conducted by a funnel plot and Begg’s and Egger’s tests.

### 3.5. Quality Assessment

Two reviewers (Miriam Dawidowicz, Sylwia Mielcarska) assessed the quality of eligible studies independently by using the Newcastle–Ottawa Quality Assessment Scale (NOS) [57]. The NOS assessed the quality of studies from the aspects of selection, comparability, and exposure, with a total score ranging from 0 to 9 points. More than 6 points was defined as high quality.

## 4. Discussion

B7H4 has been evaluated in a variety of solid tumours for its prognostic significance. In this meta-analysis, we aimed to summarise and compare the results of the published studies and extract valuable information that can be used in clinical decision making for human solid tumours. A total of 31 studies and 6357 patients were included. The results demonstrated that high B7H4 expression predicted poor OS in patients with cancers. The sensitivity analysis and publication bias proved that the results were reliable. However, heterogeneity existed among these studies. Considering the apparent heterogeneity, a subgroup analysis was performed.

The subgroup analysis indicated that a sample size, analysis method, and IHC score cut-off value did significantly reduce the heterogeneity among studies. Additionally, the subgroup analysis by cancer type revealed that B7H4 overexpression was correlated with poor OS in tumours, including CRC, GC, ESCC, and CCA, but not in PDAC, NSCLC, and RCC. Moreover, the subgroup analysis by cancer type also significantly reduced the heterogeneity within each subgroup. However, NSCLC and PDAC subgroup analyses by cancer type did not reduce heterogeneity. This might be explained by either high diversity of this cancer type, a small number of the included studies, or different methods of estimating the cut-off value for B7H4 expression.

Thus, this may suggest that cancer type was the main source of heterogeneity and that B7H4 expression may exert distinct effects in different cancer types. Studies with an IHC score cut-off value < 3 and univariate method of HR estimation did not support the relationship between high expression of B7H4 and OS. On the other hand, cut-off value was one of the heterogeneity sources. The studies where the expression of B7H4 was estimated by the most similar method had, as expected, low heterogeneity. Another factor that contributes to heterogeneity is the origin of the studied population. We did not analyse this factor in the subgroup analysis, but Qi Z-J and colleagues assessed that B7H4 was associated with worse OS in the Chinese population but not in the Japanese population [24]. The high expression of B7H4 was not significantly correlated with poor DSS, PFS, RFS, or DFS. However, the results might be inaccurate due to a relatively small number of studies that provided DSS, PFS, RFS, or DFS. Furthermore, the methods of estimating a high expression of B7H4 were highly varied in the included studies, and there were not enough studies to conduct an analysis for DSS, PFS, RFS, or DFS parameters in the subgroups to elucidate the impact of this factor.

B7H4 is not usually expressed in most normal immune cells and tissues; however, its overexpression in cancers often correlates with poor clinical outcomes and lower patient survival [9,10]. These findings were also confirmed in the previous meta-analysis. Nonetheless, there are some discrepancies between our results and the meta-analysis mentioned above. For instance, the HR in the meta-analysis conducted by Qi ZJ et al. and Song X et al. indicated poor OS in patients with PDAC, whereas our results did not support this effect of high B7H4 expression [24,26]. Secondly, patients with high B7-H4 had a significantly shorter DFS in the cohorts studied by Song X et al., whereas our analysis did not support it as well. These differences might be partially caused by including the team studies that evaluated the B7H4 expression by various methods, such as IHC and ELISA.

B7H4 is involved in tumour immunosuppressive mechanisms and is a checkpoint for inhibition [58]. Besides the prognostic value of B7H4, after PD-L1/PD-1 and CTLA-4, it is expected to be another ICB (immune checkpoint blockage) target [59]. High expression of B7H4 is associated with a poorer differentiation of tumour cells and epithelial–mesenchymal transition (EMT) of tumour cells [51,60]. Poorly differentiated tumour cells are associated with a higher presence of cancer stem cells (CSCs) in the tumour area, and this tumour phenotype is more prone to developing resistance to therapy [61]. B7H4 is expressed on CSCs, and its expression is associated with their maintenance [53,62]. Further, CSCs contribute to cancer resistance to ICI therapy [61].

Moreover, high expression of B7H4 is related to low infiltration levels of cytotoxic T lymphocytes [58,62]. The inhibition of B7H4 glycosylation has been reported to restore antitumour immunity in immune cold breast cancers [26]. These findings support the view that inhibiting the B7H4 function leads to restoring the proper T-cell function in patients with cancer. In addition, the B7H4 targeting strategy has the potential to possibly reduce the metastatic burden and tumour recurrence after therapy [61,63]. All these reasons indicate B7H4 to be an important and potent therapeutic target.

Given the limitations of this study, further well-designed studies that include evaluation of more tumour types with a larger sample size, a specifically determined cut-off value for high B7H4 expression by the IHC method, and detailed data about previously applied treatments are needed. A unified measuring method and cut-off value need to be established for prognostic analysis. The most comparable results were observed between studies that assessed B7H4 expression using an IHC score that was the percentage of positive cells and staining intensity, with a cut-off value for high expression above an IHC score of 3. In some studies, the HR and 95% CI were calculated by extracting data from Kaplan–Meier curves rather than directly from the original literature, which inevitably led to small statistical deviations. Another important matter is that researchers should provide sufficient data in their articles to enable including their results in further meta-analyses.

## 5. Conclusions

In summary, our meta-analysis provides comprehensive evidence that high B7H4 expression is associated with poor OS in solid tumours and might be used as a potential prognostic marker. However, high B7H4 expression was not related to OS in patients with PDAC, NSCLC, and RCC. The subgroup analysis that we performed helped to reduce the heterogeneity of results. The identified factors, such as cancer type, HR, and cut-off value estimation methods, significantly contributed to mitigating heterogeneity. These findings indicate the importance of evaluating different elements affecting the prognostic value of B7H4. Further studies performed to elucidate the mechanisms underlying the observed correlations and to confirm our results in larger, well-designed studies are required. Standardising methods for assessing B7H4 expression and establishing a uniform cut-off value for prognostic analysis are important steps towards improving the reliability and comparability of future studies. Therefore, more mechanistic studies are needed for further analysis.

## Figures and Tables

**Table 1:** Basic characteristics.

	Author	Year	Patient Source	Sample Size	Method	Cancer Type	B7H4 + Expression	Cut-off	Cell Types	Outcome	HR	Multivare (M)/Univare (U)	NOS
**1**	[49]	2021	China	98	IHC	CRC	0.69	>0%	tumour cells	OS/DFS	reported	U/M	8
**2**	[44]	2011	Japan	120	IHC	GC	0.94	staining 0, +/++, +++	tumour cells	OS	reported	U/M	7
**3**	[50]	2021	China	110	IHC	CRC	0.51	H score > 85	tumour cells	OS	reported	U	6
**4**	[40]	2011	China	112	IHC	ESCC	0.95	H score > 160	tumour cells	OS	reported	U/M	7
**5**	[13]	2017	China	140	IHC	CCA	0.45	Final score > 3	all types of cells	OS	reported	U/M	7
**6**	[38]	2016	China	164	IHC	HNSCC	1.00	H score > 88	all types of cells	OS	reported	U	5
**7**	[27]	2015	China	104	IHC	OS	0.7	Final score > 3	all types of cells	OS	reported	U/M	7
**8**	[30]	2020	China	50	IHC	CvC	0.32	>5%	tumour cells	OS/DFS	reported	U	7
**9**	[41]	2016	China	293	IHC	OC	0.91	Final score > 2,	tumour cells	OS/DFS	reported	U	7
**10**	[36]	2014	China	62	IHC	UCC	0.76	Final score > 4	all types of cells	OS	reported	U/M	6
**11**	[42]	2018	China	59	IHC	BC	0.91	Final score > 3	tumour cells	OS	reported	U/M	7
**12**	[28]	2020	China	152	IHC	PCa	0.67	IHC score > 1,	all types of cells	OS	reported	U/M	6
**13**	[39]	2014	China	185	IHC	CRC	0.63	Final score > 3	tumour cells	OS/DFS	reported	U	7
**14**	[53]	2018	Korea	158	IHC	ESCC	0.54	IHC score > 1,	all types of cells	OS/DFS	reported	U/M	8
**15**	[46]	2018	USA	123	IHC	NSCLC	NA	>1, 38 mediana, % of tumor cells	tumour cells	OS	reported	U	6
**16**	[46]	2018	USA	61	IHC	NSCLC	NA	>1, 59 mediana, % of tumor cells	tumour cells	OS	reported	U	6
**17**	[33]	2016	China	40	IHC	PDAC	0.75	>10%	tumour cells	OS	reported	M	7
**18**	[47]	2019	Italy	44	IHC	NSCLC	0.39	>0%	tumour cells	OS/PFS	reported	U/M	8
**19**	[47]	2019	Italy	37	IHC	NSCLC	0.4	>10%	tumour cells	OS/PFS	reported	U/M	8
**20**	[34]	2016	Greece	41	IHC	PDAC	0.39	>0%	tumour cells	OS	reported	U/M	7
**21**	[32]	2016	China	110	IHC	CCA	0.49	Final score > 3	tumour cells	OS/RFS	reported	M	8
**22**	[51]	2022	China	996	IHC	CRC	0.61	Final score > 3	tumour cells	OS	reported	M	7
**23**	[52]	2019	China	118	IHC	CRC	0.56	Final score > 3	all types of cells	OS	reported	M	7
**24**	[29]	2007	USA	814	IHC	PCa	0.8	>5% + strong intensity	tumour cells	RFS/DSS	reported	U	7
**25**	[31]	2022	China	605	IHC	CvC	0.45	≥5%	tumour cells	RFS/DSS	reported	U	7
**26**	[45]	2023	China	833	IHC	ECC	0.71	>0%	tumour cells	RFS/DSS	reported	U/M	8
**27**	[48]	2020	Japan	83	IHC	RCC	0.40	median NA	tumour cells or immune cells	OS/PFS	reported	U/M	8
**28**	[48]	2020	Japan	69	IHC	RCC	0.48	median NA	tumour cells or immune cells	OS/PFS	reported	U/M	8
**29**	[43]	2015	China	100	IHC	GC	0.71	Final score > 2		OS	reported	U/M	7
**30**	[35]	2023	Germany	68	IHC	PDAC	0.22	>1%	tumour cells	OS	pooled	U	6
**31**	[37]	2022	Germany	408	IHC	HNSCC	0.97	≤ 70% + intensity 1	tumour cells	OS	pooled	U	6

Immunohistochemistry (IHC), overall survival (OS), progression-free survival (PFS), disease-free survival (DFS), disease-specific survival (DSS), recurrence-free survival (RFS), adenocarcinoma (ADC), squamous cell carcinoma (SCC), nivolumab cohort (N), chemotherapy cohort (CTH), OS—osteosarcoma, Pca—prostate cancer, CvC—cervical cancer, CCA—cholangiocarcinoma, PDAC—pancreatic ductal adenocarcinoma, UCC—urothelial cell carcinoma, HNSCC—head and neck squamous cell carcinoma, OC—ovarian cancer, ESCC—esophageal squamous cell carcinoma, BC—breast cancer, GC—gastric cancer, ECC—endometrial cancer, NSCLC—non-squamous cell lung carcinoma, RCC—renal cell carcinoma, and CRC—colorectal cancer.

## Data Availability

The original contributions presented in the study are included in the article/Supplementary Materials, further inquiries can be directed to the corresponding author/s.

## Supplementary Materials

The following supporting information can be downloaded at: https://www.mdpi.com/article/10.3390/ijms25095045/s1.
