# Supplementary material for: Prognostic Value of B7H4 Expression in Patients with Solid Cancers: A Systematic Review and Meta-Analysis
Source: Int J Mol Sci. 2024 May 6;25(9):5045. doi: 10.3390/ijms25095045 (PMC11084412; doi:10.3390/ijms25095045)
Supplement: Supplementary file 1 [file ijms-25-05045-s001.zip › ijms-2979656-supplementary.pdf]

Table S1. Search strategy. Literature retrieval strategy(Pubmed)

1	VTCN1 [Title/Abstract]
2	B7H4 [Title/Abstract]
3	V-Set Domain-Containing T-Cell Activation Inhibitor 1 [Title/Abstract]
4	B7x [Title/Abstract]
5	B7-H4 [Title/Abstract]
6	1 OR 2 OR 3 OR 4 OR 5
7	Carcinoma [Title/Abstract]
8	Carcinoma[MeSH]
9	Neoplasms[Title/Abstract]
10	Neoplasms[MeSH]
11	cancer [Title/Abstract]
12	neoplasia [Title/Abstract]
13	Tumor [Title/Abstract]
14	malignancy [Title/Abstract]
15	malignant neoplasm (Full text)
16	7 OR 8 OR 9 OR 10 OR 11 OR 12 OR 13 OR 14 OR 15
17	Prognosis [MeSH]
18	Prognosis [Title/Abstract]
19	Survival Analysis [MeSH]
20	Survival Analysis [Title/Abstract]
21	Prognostic [Title/Abstract]
22	Treatment Outcome [MeSH]
23	Treatment Outcome [Title/Abstract]
24	Fatal Outcome [MeSH]
25	Fatal Outcome [Title/Abstract]
26	Mortality [MeSH]
27	Mortality [Title/Abstract]
28	17 OR 18 OR 19 OR 20 OR 21 OR 22 OR 23 OR 24 OR25 OR 26 OR 27
29	6 AND 16 AND 28

Table S2. Search strategy. Literature retrieval strategy(Embase)

1	VTCN1 [Title/Abstract]
2	B7H4 [Title/Abstract]
3	V-Set Domain-Containing T-Cell Activation Inhibitor 1 [Title/Abstract]
4	B7x [Title/Abstract]
5	B7-H4 [Title/Abstract]
6	1 OR 2 OR 3 OR 4 OR 5
7	Carcinoma [Title/Abstract]
8	Neoplasms[Title/Abstract]
9	cancer [Title/Abstract]
10	neoplasia [Title/Abstract]
11	Tumor [Title/Abstract]

12	malignancy [Title/Abstract]
13	malignant neoplasm [Title/Abstract]
14	7 OR 8 OR 9 OR 10 OR 11 OR 12 OR 13
15	Prognosis* [Title/Abstract]
16	Survival Analysis [Title/Abstract]
17	Treatment Outcome [Title/Abstract]
18	Fatal Outcome [Title/Abstract]
19	Mortality [Title/Abstract]
20	15 OR 16 OR 17 OR 18 OR 19
21	6 AND 14 AND 20

Table S3. Search strategy. Literature retrieval strategy (Cochrane library)

1	VTCN1 [Title Abstract Keyword]
2	V-Set Domain-Containing T-Cell Activation Inhibitor 1 [Title Abstract Keyword]
3	B7H4 [Title Abstract Keyword]
4	B7x [Title Abstract Keyword]
5	B7-H4 [Title Abstract Keyword]
6	1 OR 2 OR 3 OR 4 OR 5
7	Carcinoma [Title Abstract Keyword]
8	Neoplasms[Title Abstract Keyword]
9	cancer [Title Abstract Keyword]
10	neoplasia [Title Abstract Keyword]
11	Tumor [Title Abstract Keyword]
12	malignancy [Title Abstract Keyword]
13	malignant neoplasm [Title Abstract Keyword]
14	7 OR 8 OR 9 OR 10 OR 11 OR 12 OR 13
15	Prognosis* [Title Abstract Keyword]
16	Survival Analysis [Title Abstract Keyword]
17	Treatment Outcome [Title Abstract Keyword]
18	Fatal Outcome [Title Abstract Keyword]
19	Mortality [Title Abstract Keyword]
20	15 OR 16 OR 17 OR 18 OR 19
21	6 AND 14 AND 20

Table S4.

	**Study**	**Selection**				**Comparability**	**Exposure**			**Scores**
		Representativeness of the exposed cohort	Selection of the nonexposed cohort	Ascertainment of exposure	Demonstration that outcome of interest was not present at start of study	Comparability of cohorts on the basis of the design or analysis	Assessment of outcome	Was follow-up long enough for outcomes to occur	Adequacy of follow up of cohorts	
1	Feng Y *et al,* 2021	«	«	«	-	««	«	«	«	8

2	Arigami T *et al,* 2011	«	«	«	-	««	-	«	«	7
3	Ding S et al, 2021	«	«	«	-	«	-	«	«	6
4	Chen L *et al,* 2011	«	«	«	-	««	-	«	«	7
5	Xie N *et al,* 2017	«	«	«	-	««	-	«	«	7
6	Wu L *et al,* 2016	«	«	«	-	«	-	-	«	5
7	Dong Q *et al,* 2015	«	«	«	-	««	-	«	«	7
8	Zong L *et al,* 2020	«	«	«	-	«	«	«	«	7
9	Liang L *et al,* 2016	«	«	«	-	«	«	«	«	7
10	Fan M *et al,* 2014	«	«	«	-	««	-	-	«	6
11	Wang L *et al,* 2018	«	«	«	-	««	-	«	«	7
12	Li H *et al,* 2020	«	«	«	-	««	-	-	«	6
13	Liang M *et al,* 2014	«	«	«	-	«	«	«	«	7
14	Piao L *et al,* 2018	«	«	«	-	««	«	«	«	8
15	Parra ER *et al,* 2018	«	«	«	-	«	-	«	«	6
16	Parra ER *et al,* 2019	«	«	«	-	«	-	«	«	6
17	Xu H *et al,* 2016	«	«	«	-	««	-	«	«	7
18	Genova C *et al,* 2019	«	«	«	-	««	«	«	«	8
19	Genova C *et al,* 2020	«	«	«	-	««	«	«	«	8
20	Tsiaousidou A *et al,* 2016	«	«	«	-	««	-	«	«	7
21	Zhao X *et al,* 2016	«	«	«	-	««	«	«	«	8
22	Yan X *et al,* 2022	«	«	«	-	««	-	«	«	7
23	Cao H *et al,* 2019	«	«	«	-	««	-	«	«	7
24	Zang X, 2007	«	«	«	-	«	«	«	«	7
25	Zong L *et al,* 2022	«	«	«	-	«	«	«	«	7
26	Zong L *et al,* 2023	«	«	«	-	««	«	«	«	8
27	Jikuya R *et al,* 2020	«	«	«	-	««	«	«	«	8
28	Jikuya R *et al,* 2021	«	«	«	-	««	«	«	«	8
29	Geng Y *et al,* 2015	«	«	«	-	««	-	«	«	7
30	Loch F N *et al,* 2023	«	«	«	-	«	-	«	«	6
31	Borgmann M *et al,* 2022	«	«	«	-	«	-	«	«	6

Figure S1.

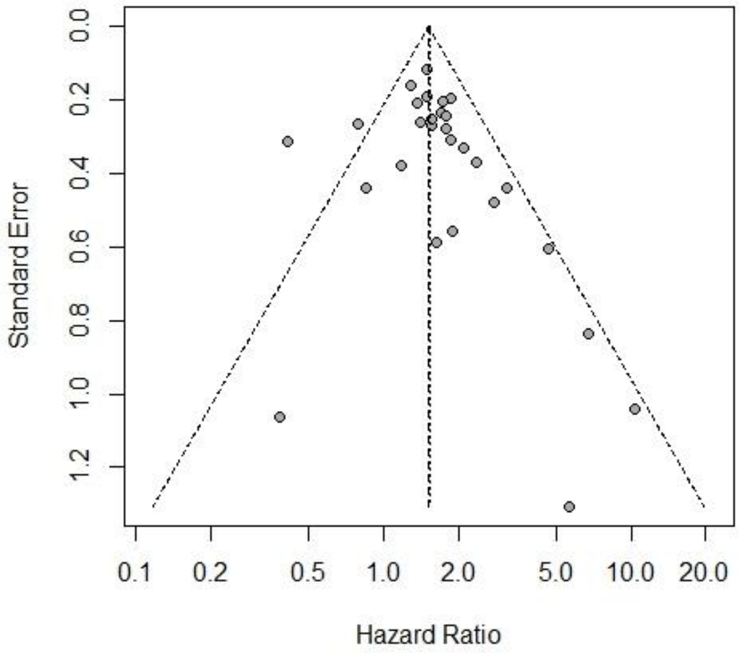

.

Figure S1. Begg's funnel plots for assessing potential publication bias on the relationship between B7H4 expression and overall survival in patients with solid tumors. Egger test *p* value= 0.6185, Begg's test *p* value=0.9104 (*p*>0.05- Bias was not detected in Egger's and Begg;s tests)

Figure S2.

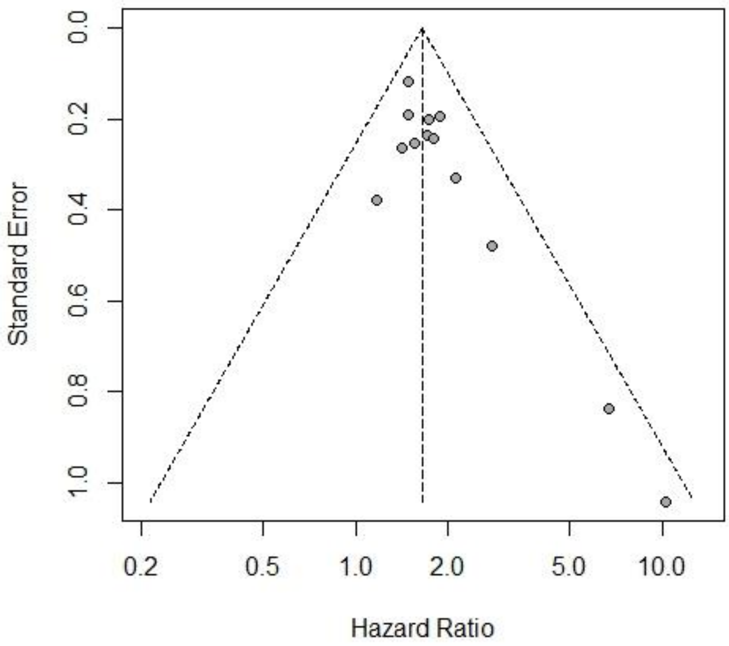

Figure S2. Begg's funnel plots for assessing potential publication bias on the relationship between B7H4 expression and overall survival in patients with gastrointestinal malignancies. Egger test *p* value= 0.0059, Begg's test *p* value=0.0381 (*p*<0.05- Bias was detected in Egger's and Begg;s tests)

Figure S3

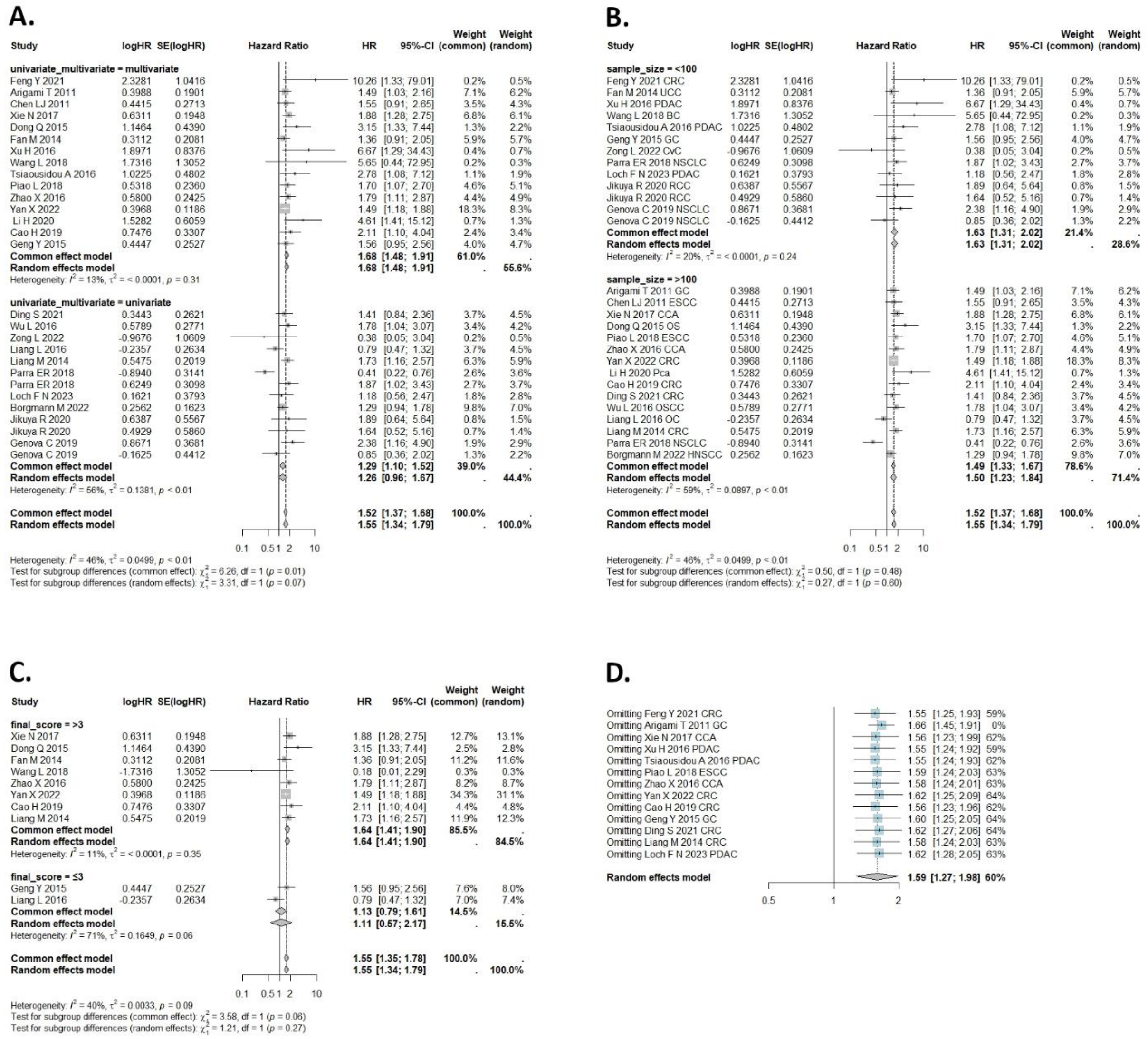

Figure S3. Subgroup analysis for the relationship between B7H4 and overall survival **(A)** Grouped by different methods to estimate HR. **(B)** Grouped by sample size **(C)** Grouped by final score in IHC assessment. **(D)** One-leave meta-analysis for investigating the effects of particular studies on the association between B7H4 expression and overall survival in gastrointestinal cancers.

Table S5.

**Terms**	**Definition**
OS	Overall survival (OS) was defined as the duration between time 0 to the day when patients succumbed, underwent truncation or the final follow up.
DSS	Disease-Specific Survival (DSS) refers to deaths caused specifically by a particular disease.
DFS	Disease-free survival (DFS) The time from random assignment to cancer recurrence or death from any cause..
PFS	Progression-free survival (PFS), defined as the time from random assignment in a clinical trial to disease progression or death from any cause
RFS	Relapse-free survival (RFS) The time from treatment of disease to any event, irrespective of cause, except for any second primary cancers
